# Image-domain assessment and quantitative evaluation of acoustic cavitation using singular-value decomposition and magnitude-based spectral gating of B-mode imaging^☆^

**DOI:** 10.1016/j.ultsonch.2026.108114

**Published:** 2026-10-13

**Authors:** Xinyue Gong, Zijun Liu, Guofeng Zhang, Shuyuan Gu, Yifei Zhu, Honghui Xue, Qinsong Xu, Jun Wu, Juan Tu

**Affiliations:** a Department of Acoustics, School of Physics, Jiangsu Key Laboratory for Cardiovascular Information and Health Engineering Medicine, Nanjing University, Nanjing 210093, China; b School of IOT and AI, Wuxi Vocational Institute of Commerce, Wuxi 214153, China; c Dept. of EME, FST, University of Macau, Avenida da Universidade, Taipa, Macau; d Department of Geriatric Cardiology, Jiangsu Provincial Key Laboratory of Geriatrics, The First Affiliated Hospital of Nanjing Medical University, 300 Guangzhou Rd, Nanjing 210029, China

**Keywords:** Cavitation monitoring and evaluation, Singular value decomposition, Magnitude-based spectral gating, Stripe-artifact suppression, B-mode ultrasound imaging

## Abstract

Acoustic cavitation is a transient and spatially heterogeneous process that governs energy deposition and mechanical effects during high-intensity focused ultrasound (HIFU) exposure. Active B-mode imaging is attractive for monitoring because it is readily available on clinical ultrasound systems; however, therapy-imaging coupling can introduce drifting stripe artifacts that overlap with cavitation backscatter and compromise gray-level thresholding and region of interest (ROI) based quantification. Here we present an image-domain B-mode framework for cavitation-associated feature separation and spatiotemporal quantification based on singular-value decomposition (SVD) and magnitude-based spectral gating. B-mode sequences are rearranged into a spatiotemporal matrix, decomposed into singular components, and reconstructed into full-field maps of cavitation-associated image features from finite frame stacks. Highly coherent components are assigned to quasi-steady HIFU-related echoes and background scattering, whereas candidate cavitation components are selected according to their spectral-magnitude distribution, allowing stripe components observed in the present B-mode sequences to be suppressed while intermittent candidate cavitation-associated image fluctuations are retained. Experiments in egg-white polyacrylamide gels and *ex vivo* bovine liver showed that the proposed method suppressed stripe artifacts, improved frame-to-frame consistency, and detected cavitation onset earlier than conventional grayscale thresholding. The image-derived cavitation area increased with both acoustic pressure and pulse length and followed the trends observed in post-exposure axial damage morphology. This unsupervised method requires neither training data nor manual ROI selection, and therefore provides a practical image-based metric for retrospective visualization and quantification of cavitation-associated features during therapeutic ultrasound exposures.

## 1 Introduction

Ultrasound-mediated cavitation plays a key role in many power-ultrasound processes [1], [2], [3]. In therapeutic ultrasound, such as histotripsy [4], [5], blood–brain barrier (BBB) opening [6], [7], localized drug and gene delivery [8], [9], and other cavitation-assisted applications [10], [11], the growth and collapse of bubbles can alter local pressure fields, enhance microstreaming and mechanical stress, and modify the biological response to exposure [12], [13]. The same phenomenon is also a major source of uncertainty, because cavitation may enhance treatment efficiency, but uncontrolled bubble activity can shift the exposure from a desired mechanical effect to tissue disruption or off-target damage [14], [15]. Recent studies have further emphasized that cavitation dynamics are strongly governed by the surrounding medium, boundary conditions, and external physical fields, with substantial implications for biomedical and engineering applications [16], [17]. These findings further highlight the need for monitoring approaches capable of tracking the transient and spatially heterogeneous behavior of cavitation under different exposure environments. For this reason, cavitation should be treated not as a binary event but as a dynamic field variable whose onset, spatial extent, persistence and decay need to be monitored.

Several approaches have been developed to monitor cavitation, including passive cavitation detection (PCD) [18], [19], [20], [21], array-based acoustic mapping [22], active ultrasound imaging [23], [24] and high-frame-rate B-mode or Doppler-based visualization [25], [26]. Passive methods provide direct access to cavitation emissions, but they often require compromises among spatial localization, temporal resolution, system integration and computational burden [29]. Active B-mode imaging is easier to integrate with ultrasound platforms and provides intuitive spatial information. However, B-mode sequences acquired during exposure are contaminated by acoustic leakage, stripe artifacts, reverberation and tissue-background fluctuations [18], [19], [30]. These signals can overlap with cavitation-related backscatter, making intensity thresholding and fixed-region measurements unreliable.

For quantitative cavitation analysis, a monitoring method should distinguish bubble-cloud activity from structured interference and should provide a metric that can be tracked throughout exposure. Hyperechoic changes in B-mode images indicate the presence of scattering structures, but echogenicity alone does not specify the spatial distribution or intensity of mechanically relevant cavitation. A useful readout should therefore be full-field, time resolved and robust to stripe contamination. It should also minimize operator dependence, because manual region-of-interest (ROI) selection can bias area-time estimates and limit reproducibility.

Singular-value decomposition (SVD) provides a natural basis for this task because it decomposes an image sequence into components with different spatial and temporal coherence [31], [32], [33], [34]. Currently, SVD has been widely used for clutter suppression in ultrafast Doppler imaging. In cavitation monitoring, the underlying signal classes also differ in coherence: quasi-steady HIFU-related echoes are spatially stable, drifting stripe artifacts have structured temporal periodicity, and cavitation backscatter is intermittent and spatially localized. These differences suggest that SVD can be combined with temporal spectral analysis to distinguish cavitation-related components from coherent interference.

The aim of this study was to develop and evaluate an SVD-based framework for separating and quantifying candidate cavitation-associated image features during active B-mode imaging. The method exploits differences in spatiotemporal coherence and temporal spectral characteristics among background echoes, exposure-induced interference, and intermittent backscatter potentially associated with cavitation. Components identified by spectral gating are reconstructed into full-field maps of candidate cavitation-associated features, enabling the temporal rise, spatial expansion, persistence, and decay of the reconstructed image features to be tracked without a predefined region of interest. The significance of the proposed framework lies in providing a more robust and operator-independent image-domain readout from standard B-mode sequences under structured interference, thereby facilitating retrospective treatment assessment and relative comparisons across exposure conditions. The framework was evaluated in transparent egg-white polyacrylamide gels and *ex vivo* bovine liver under controlled acoustic pressures and pulse lengths. Specifically, we assessed whether the proposed method could suppress structured stripe artifacts, produce an earlier rise in the image-derived cavitation-area curve than conventional grayscale thresholding, and yield a feature-area metric that varied systematically with acoustic pressure and pulse length and showed trends paralleling post-exposure damage morphology. Accordingly, the reconstructed outputs are interpreted as image-domain cavitation-associated measurements, while synchronized acoustic measurements were not available for direct acoustic calibration in the present dataset.

## 2 Materials and methods

### 2.1 Algorithmic framework and signal separation model

In active ultrasound monitoring sequences acquired during HIFU exposure, echoes originating from different physical sources exhibit distinct spatiotemporal coherence patterns. Quasi-steady driving echoes are closely related to the acoustic-field structure and typically appear as highly coherent components with stable spatial localization and slow temporal variation. Stripe artifacts, which mainly arise from multipath propagation and phase superposition, form regular textures that drift periodically or quasi-periodically over time. By contrast, cavitation-related bubble activity can produce temporally evolving hyperechoic features in B-mode images [23], and in the present sequences these features appeared intermittently with lower temporal coherence and rapid spatiotemporal variation.

Based on these physical differences, we treat the B-mode image sequence as a spatiotemporal signal ensemble generated by the superposition of multiple echo mechanisms and develop a cavitation separation framework that combines SVD with spectral-magnitude analysis. The workflow includes spatiotemporal matrix construction, SVD, spectral-magnitude-based discrimination, and component-wise reconstruction. As illustrated in Fig. 1, signals of different origins exhibit marked differences in singular-value energy distribution, spatial correlation, and temporal consistency. By exploiting these differences, SVD can effectively disentangle tissue/background echoes, stripe artifacts, and cavitation signals without relying on predefined spatial locations or empirical intensity thresholds. The proposed pipeline is unsupervised and does not require manual outlining of the cavitation region. In the present implementation, SVD was performed on finite batches of B-mode frames rather than on a causal sliding window scheme. At a frame rate of 30 frames/s, each stack corresponded to a 4-s temporal interval.

**Fig. 1:**
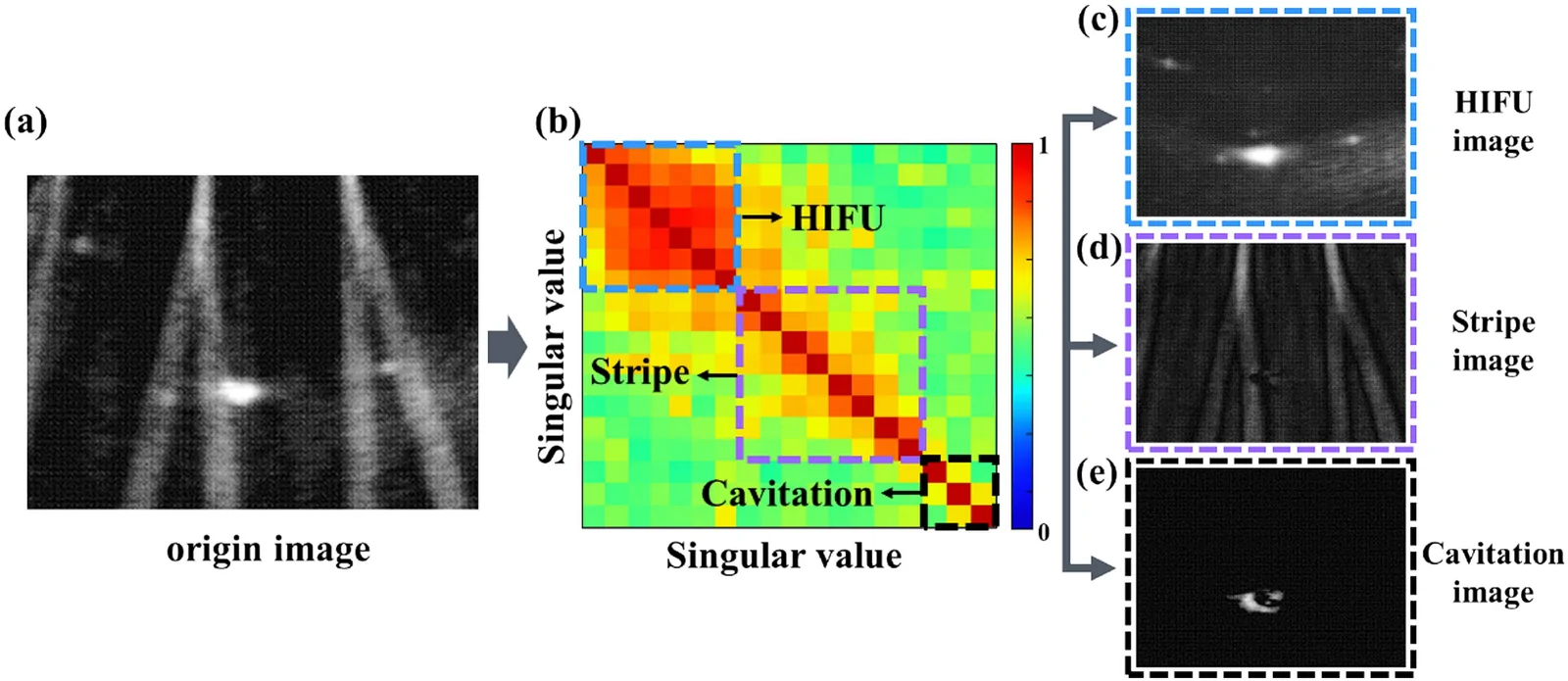
Schematic of the SVD-based cavitation-signal separation framework. B-mode sequences acquired during HIFU exposure are transformed into a spatiotemporal matrix and decomposed by SVD. Spectral-magnitude analysis of temporal singular components is used to separate HIFU-related echoes, stripe artifacts, and cavitation-related signals for full-field cavitation mapping.

Assume that the ultrasound system acquires a sequence of B-mode images at a constant frame rate. Each frame has a size of $n_{x}\times n_{z}$ and can be reshaped into a vector of size $1\times \left(n_{x}n_{z}\right)$. All frames are stacked in temporal order to form an image spatiotemporal matrix S. Following the conventional spatiotemporal SVD formulation used for ultrasound sequence decomposition and clutter filtering [31], [32], [33], [34], the SVD of matrix S is expressed as:

(1) $${S=U\Delta V}^{*},$$

Here, $\Delta =\text{diag}\left(\sigma_{1},\sigma_{2},\ldots ,\sigma_{i}\right)$ is a diagonal matrix of size $\left(n_{x}\times n_{z},n_{t}\right)$, whose diagonal elements, $\sigma_{i}$ are the singular values arranged in descending order. Matrix U is an orthonormal matrix along the spatial dimension and describes the spatial variation of each component, whereas matrix V is an orthonormal matrix along the temporal dimension and represents the temporal distribution of each component. Their dimensions are $\left(n_{x}\times n_{z},n_{x}\times n_{z}\right)$ and $\left(n_{t},n_{t}\right)$ respectively.

Each singular component $u_{i}\sigma_{i}v_{i}^{*}$ represents a rank-1 term of the decomposition. Signals with strong spatiotemporal coherence, such as tissue echoes and structured HIFU-related interference, are generally represented by components associated with larger singular values, whereas intermittent image fluctuations tend to be distributed across less coherent components. In the present datasets, the first singular component consistently exhibited the largest singular value, a spatially stable focal pattern, and the lowest temporal variability, and was therefore operationally assigned as the dominant HIFU-related coherent component. This assignment was based on the observed characteristics of the present datasets rather than an a priori assumption; under different exposure or acquisition conditions, HIFU-related signal content may extend across multiple leading singular components. Stripe artifacts show periodicity and strong spatiotemporal correlation and are usually distributed among intermediate singular values, as highlighted by the purple dashed box in Fig. 1(b). By contrast, in the present datasets, candidate cavitation-associated image fluctuations were spatially localized and temporally intermittent. Their assignment was subsequently refined using the image-domain temporal spectral criterion described below rather than by singular-value rank alone.

In practice, selecting components solely on the basis of singular-value magnitude is not sufficiently robust for distinguishing cavitation from stripe interference, especially when their energies are comparable. To address this limitation, we further analyzed the temporal singular components in the frequency domain. Specifically, an FFT was applied to the temporal trace of each reconstructed singular component, obtained by spatially averaging the component over the image field. Component selection was performed using magnitude-based spectral gating. A low-frequency spectral-magnitude fraction, $A_{r}\left(i\right)$, was calculated as:

(2) $$A_{r}\left(i\right)=\frac{\sum_{k=1}^{3}\left|F_{i}\left(k\right)\right|}{\sum_{k=0}^{N/2-1}\left|F_{i}\left(k\right)\right|},\;A_{r}\left(i\right)>\tau ,\;\tau =0.07,$$

Here, $F_{i}\left(k\right)$ denotes the FFT spectrum of the i-th temporal component, k denotes the zero-based frequency bin, and N is the stack length. For the spectra shown in Figs. 3e-3g, the analyzed sequence comprised 120 consecutive B-mode frames acquired at 30 frames/s, giving a frequency-bin spacing of 0.25 Hz. The low-frequency non-DC band was therefore defined as bins *k* = 1, 2 and 3, corresponding to 0.25, 0.5 and 0.75 Hz, and the spectral-magnitude fraction threshold was set to τ=0.07. The order of magnitude of this fractional threshold was informed by previous spectral cavitation studies. For example, George et al. employed a 10% limit of normalized voltage spectral density as an operational threshold for identifying inertial cavitation in spectral measurements [35]. Although the normalized spectral quantity used in that study is not mathematically equivalent to the low-frequency spectral-magnitude fraction defined here, it provided an empirical order-of-magnitude reference for selecting the initial threshold range.

Candidate values around this range were subsequently examined during preliminary analysis of the present B-mode sequences. Lower thresholds increased the retention of weakly varying components but also admitted more quasi-steady background and interference-dominated contributions, whereas higher thresholds provided stronger rejection but increasingly suppressed weak and intermittent candidate cavitation-associated fluctuations. Accordingly, τ=0.07 was selected as a practical operating point that provided a reasonable trade-off between feature retention and interference rejection under the present acquisition conditions.

Because no synchronized acoustic or optical cavitation reference was available, this value was not treated as a statistically optimized detection threshold. To evaluate its influence on the reconstructed image-domain readout, we further performed a retrospective sensitivity analysis by varying τ from 0.056 to 0.084 (±20% around the reference value) while keeping q=3 (i.e., *k* = 1 to 3), and by separately varying the highest included non-DC bin from q=2 to q=5 at τ=0.07. As expected from the gating formulation, increasing τ progressively reduced or preserved the retained-component count, whereas increasing the highest included non-DC bin progressively increased or preserved it (Figs. S1a and b). The downstream reconstructed foreground measurements showed additional sequence-dependent variation because they were obtained after component reconstruction and image-domain segmentation (Figs. S1c and d).

The spectral gate operates on the sampled temporal intensity spectrum of each singular component, with the frequency of bin k given by $f_{k}=kf_{s}/N$, where $f_{s}$ is the B-mode frame rate and N is the stack length. The criterion characterizes the temporal spectral distribution of an image-derived component. Therefore, a stripe-, mains-, scanner-, or PRF-related component may be retained if its sampled, aliased, or modulated spectrum overlaps the accepted band. Conversely, a cavitation-associated image component may be rejected if its temporal spectral content falls predominantly outside that band.

Accordingly, components satisfying $A_{r}\left(i\right)>\tau$ were retained as candidate cavitation-associated image components. By combining the spectral-magnitude criterion with singular-value-based screening, the candidate component set $S_{\text{cav}}$ was determined. Following the standard component-wise reconstruction of SVD-filtered ultrasound data [31], [32], [33], [34], the corresponding image matrix was reconstructed as:

(3) $$S_{\text{cav}}=\sum_{i\in S_{\text{cav}}}u_{i}\sigma_{i}v_{i}^{*}.$$

The reconstructed cavitation image is shown in Fig. 1(e). In addition, individual components can be reconstructed separately: the HIFU main-signal image is obtained as $S_{\text{HIFU}}=u_{1}\sigma_{1}v_{1}^{*}$, as illustrated in Fig. 1(c), while the stripe artifact image is reconstructed by $S_{\text{stripe}}=S-S_{\text{HIFU}}-S_{\text{cav}}$ with the resulting stripe signal image shown in Fig. 1(d).

### 2.2 Experimental setup

An optically transparent polyacrylamide gel phantom containing egg white was used as the tissue-mimicking medium. For each 100 mL of precursor solution, the mixture contained 43.1 mL of degassed water, 30 mL of egg white, 26.2 mL of 40% (v/v) acrylamide aqueous solution, 0.5 mL of 10% ammonium persulfate (ammonium peroxodisulfate) solution, and 0.2 mL of N,N,N′,N′-tetramethylethylenediamine (TEMED). The formulation was adapted from Takegami et al [36].

The egg white was filtered three times through a stainless-steel mesh with a pore size of 0.045 mm and then centrifuged at 3000 rpm for 10 min. The supernatant was refrigerated until it cooled to 43 °C to avoid protein denaturation caused by the exothermic heat released during polymerization. The cooled egg-white solution was then added to a thoroughly mixed solution containing 40% (w/v) acrylamide (Amresco Inc., USA) and 1.3% (w/v) N,N′-methylenebisacrylamide and stirred until homogeneous. An ammonium persulfate solution (Amresco Inc., USA) was subsequently added, followed by degassed water, and the mixture was stirred again to ensure uniformity. Finally, TEMED (Amresco Inc., USA) was added to accelerate polymerization. The precursor solution was poured into a mold and allowed to gel at room temperature for 30 min, forming a transparent phantom. Under ultrasound exposure, the egg-white component turned white and opaque once the temperature rose to approximately 58 °C. A freshly prepared phantom was used in each experiment.

*Ex vivo* tissue samples were obtained from freshly harvested bovine liver collected on the day of slaughter from a local abattoir. The liver was cut into uniform blocks measuring 10 × 10 × 4 cm^3^, degassed, and mounted in a sample holder. The samples were then immersed in physiological saline, and HIFU irradiation was applied with the acoustic focus positioned 1.5 cm beneath the sample surface [37].

As shown in Fig. 2, the experimental system consisted of a focused HIFU transducer (1.12 MHz center frequency, 10 cm aperture, and 10 cm focal length), an arbitrary waveform generator (Agilent 33250A, USA) that produced pulsed signals at 1.12 MHz with a pulse repetition frequency (PRF) of 100 Hz, and a power amplifier (E&I 2200L, USA) together with an impedance-matching network to drive the transducer. B-mode videos were acquired at 30 frames s^−1^ using a portable clinical ultrasound system (ECARE EC50A, China) equipped with a linear-array probe (L12-5L40N-3, ECARE, China). The input data were exported display B-mode videos rather than raw RF or IQ data. The frames were therefore processed in the log-compressed image domain after grayscale conversion. The SVD and spectral gating were applied to pixel intensities in this image domain. The SVD analysis itself was performed on finite frame stacks after acquisition and was not implemented as a real-time causal sliding-window process in the present study. A three-axis motorized positioning stage (Newport ESP7000, USA) was used to align the imaging plane with the HIFU focal zone. A LabVIEW-based control module (NI Corp., TX, USA) synchronized waveform output, ultrasound acquisition, and stage motion through TTL triggering.

**Fig. 2:**
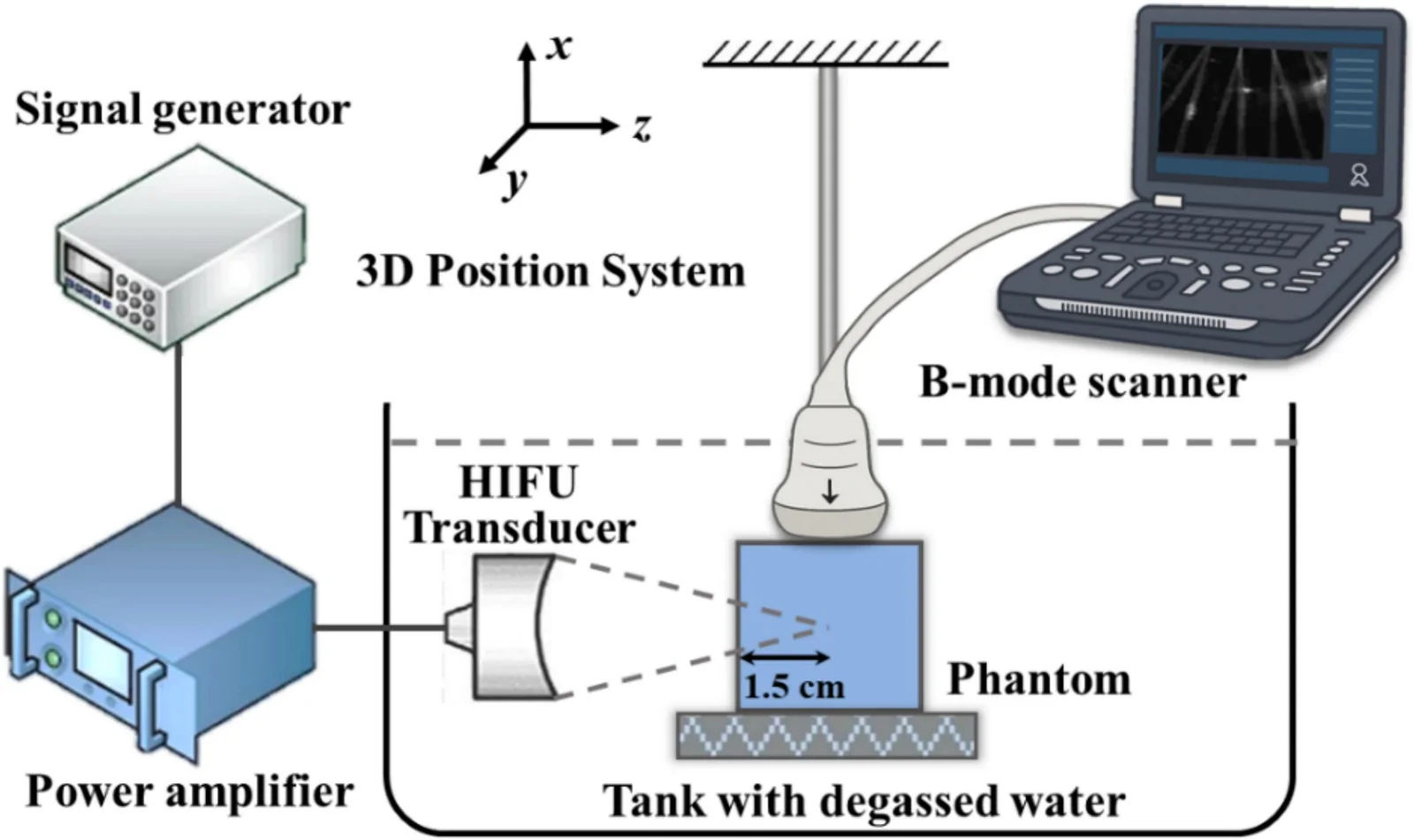
Experimental system for active B-mode monitoring of HIFU-induced cavitation. A focused 1.12-MHz HIFU transducer generated cavitation in gel phantoms and *ex vivo* liver samples, while a portable ultrasound system acquired B-mode videos at 30 frames s^−1^. System synchronization was achieved using a LabVIEW-based TTL control module.

To systematically evaluate the dependence of cavitation behavior on acoustic excitation, two parameter-controlled experiments were performed. In the first experiment, the pressure amplitude was fixed at 7.50 MPa and the pulse length was varied to 2000, 3000, 4000, and 6000 cycles. In the second experiment, the pulse length was fixed at 2000 cycles and the pressure amplitude was varied to 7.50, 8.75, and 9.92 MPa. All experiments were conducted under identical imaging settings and acoustic-field geometry. Each acquisition lasted 20 s, and image processing was performed over the full ultrasound field of view. HIFU-induced hyperechoic changes in B-mode imaging have previously been correlated with cavitation activity using synchronized passive cavitation detection [23], [41] and have been used for quantitative monitoring of cavitation-bubble evolution [43]. In the present experiments, post-exposure macroscopic damage morphology was additionally used as an independent endpoint for evaluating the spatial and exposure-dependent trends obtained from the SVD-derived B-mode measurements.

### 2.3 Morphological characterization of cavitation-induced damage and three-dimensional reconstruction

Following HIFU exposure, the tissue-mimicking phantoms and *ex vivo* liver samples were physically sectioned through the treated region, and digital photographs of the exposed damage cross-sections were acquired to document the macroscopic damage morphology. These post-exposure observations were used as an independent morphological endpoint for comparison with the cavitation-associated regions identified from the B-mode image sequences. Damage morphology was represented and quantified consistently using axial cross-sections.

In all experiments, cavitation-associated regions were first obtained by applying SVD-based separation to the axial B-mode images. In the resulting cavitation images, high-intensity regions represented the main spatial distribution of cavitation-associated hyperechoic features. These regions were binarized and subjected to connected-component analysis, and the number of pixels was extracted as an image-derived cavitation-area metric. This pixel-based metric was used to construct feature-area-time curves and to compare the temporal evolution and relative magnitude of the image-derived metric under different acoustic conditions.

On the basis of the two-dimensional analysis, three-dimensional reconstruction was further performed to visualize the macroscopic spatial morphology of cavitation-induced damage under different excitation conditions and to facilitate comparison with the directly observed post-exposure damage photographs. The reconstruction relied exclusively on axial two-dimensional images and did not introduce additional radial imaging information. Specifically, the damage region in each axial slice was first segmented as the foreground, and pixel-to-physical scale calibration was performed using the embedded spatial scale markers. The processed axial slices were then fed into a generative three-dimensional reconstruction pipeline to generate volumetric damage structures that were morphologically consistent with their corresponding two-dimensional representations.

Three-dimensional meshes were generated using Meshy.ai [38], [39], [40]. The reconstruction pipeline comprised the following stages: (1) semantic and appearance encoding, in which the input two-dimensional images together with the damage foreground masks and scale markers were processed by a visual encoder to extract semantic and appearance priors; (2) multi-view diffusion generation, in which a multi-view-consistent diffusion model inferred mutually consistent front, back, left, right, top, and bottom views from one or a small number of input images, thereby completing occluded or unobserved perspectives; (3) implicit-field reconstruction, in which large reconstruction models (LRMs) combined with neural radiance field (NeRF) representations or Gaussian splatting were used to lift the multi-view images into a continuous volumetric or radiance-field representation; (4) geometry extraction and meshing, in which an approach analogous to Deep Marching Tetrahedra (DMTet) was used to extract isosurfaces from the implicit field, followed by topological optimization to generate high-quality triangular meshes; and (5) physically based rendering (PBR) texturing, in which albedo, normal, and roughness maps were estimated to produce stable surface appearance under physically based rendering.

The three-dimensional reconstructions presented here were not intended to recover the true anatomical geometry of the tissue. Instead, they were used as a morphological complement to the two-dimensional cavitation-area analysis. The derived three-dimensional volume metrics were used only for relative trend assessment and cross-parameter comparison under different ultrasonic conditions. Their physical significance lies in reflecting the overall spatial extent of cavitation-induced damage rather than providing anatomical ground truth. It should be noted that this generative three-dimensional reconstruction was used only for morphological visualization and relative trend assessment and did not constitute a physics-based numerical simulation of cavitation dynamics.

### 2.4 Statistical analysis

To quantify the effects of acoustic pressure and pulse length on the image-derived cavitation area, all area-based metrics were calculated from cavitation maps obtained after SVD-based separation. The extracted cavitation regions were binarized using the same thresholding strategy across all experimental conditions, and the number of connected foreground pixels in each analyzed image was used as the image-derived cavitation-area metric. Statistical comparisons were performed separately for the pulse-length experiment and the acoustic-pressure experiment, and the gel phantom and *ex vivo* liver datasets were analyzed independently. Continuous data are presented as violin plots overlaid with box plot, where the center line indicates the median, the box limits indicate the interquartile range (IQR), and the whiskers indicate 1.5 × IQR. Time-resolved cavitation-area curves are reported as mean ± SD, with the shaded band representing the corresponding dispersion measure.

Before statistical comparison, normality within each group was assessed using the Shapiro-Wilk test, and homogeneity of variance across groups was evaluated using Levene’s test. For datasets satisfying these assumptions, differences among pulse-length conditions (2000, 3000, 4000, and 6000 cycles at 7.50 MPa) and among acoustic-pressure conditions (7.50, 8.75, and 9.92 MPa at 2000 cycles) were evaluated separately using one-way analysis of variance (ANOVA). When a significant overall group effect was identified, Bonferroni-adjusted pairwise comparisons were performed to control for multiple testing. A two-sided P<0.01 was considered statistically significant.

The 3D reconstructions were used to visualize relative morphological changes across conditions rather than to recover anatomical ground truth. To control the uncertainty associated with the generative reconstruction, repeated reconstructions were performed for each parameter setting, and the coefficient of variation (CV) of the reconstructed volume was required to be < 5%. Volumetric changes were further cross-checked against axial two-dimensional metrics (pixel area and axial length). Directional consistency was required, and the relative deviation between reconstructed volume and axial length was constrained to < 10%. The reconstructed volume was used only for cross-parameter trend interpretation and was not included in statistical significance testing.

## 3 Results

Based on the proposed SVD decoupling and spectral-analysis framework, the acquired B-mode image sequences were processed and representative results from a single frame are shown in Fig. 3. The original B-mode image is presented in Fig. 3a. As shown in Figs. 3b-d, the SVD-based approach effectively separates the HIFU driving signal, the cavitation signal, and the stripe-interference component.

In the present dataset, the first singular component exhibited the largest singular value, a spatially stable focal pattern, and minimal temporal variation. It was therefore operationally identified as the dominant HIFU-related coherent component for subsequent reconstruction (Fig. 3b), rather than being assumed a priori to contain all HIFU-related signal content. In Fig. 3b, this component appears as a spatially stable high-intensity focal region with a relatively clean background. Components whose spectral energy exceeded the predefined threshold were then summed to reconstruct the cavitation-related component, yielding a flickering bubble-cloud pattern concentrated around the focus (Fig. 3c) whose location was consistent with the HIFU focal region. The stripe-artifact component was subsequently obtained as the residual after subtracting the HIFU-driven and cavitation-related components from the original data. The extracted artifacts showed the expected drifting banded pattern, and a representative example from the same frame is displayed in Fig. 3d.

The temporal amplitude spectra in Figs. 3e-3g illustrate differences among the image-derived components under the present acquisition conditions. The dominant coherent component shows a strong contribution near zero frequency, while the candidate cavitation-associated component exhibits additional low-frequency variation. The stripe-associated component shows a prominent peak at approximately 14 Hz, toward the upper end of the sampled temporal frequency range. These observations support the empirical spectral distinction in this example, while the limitations associated with spectral overlap are discussed in Section 4.6.

To verify the temporal consistency of the decomposition and the visual interpretability of cavitation expansion, four consecutive time points acquired under the same acoustic dose were selected for frame-by-frame visualization (Fig. 4). The top row shows the original B-mode images, in which a hyperechoic region near the focus progressively enlarges over time and tends to extend along the beam axis. The bottom row presents the SVD-separated cavitation-only images, from which the steady HIFU scattering and stripe artifacts have been removed. In these processed images, the cavitation cluster exhibits monotonic spatial expansion over time, together with improved boundary continuity and increased internal intensity.

**Fig. 3:**
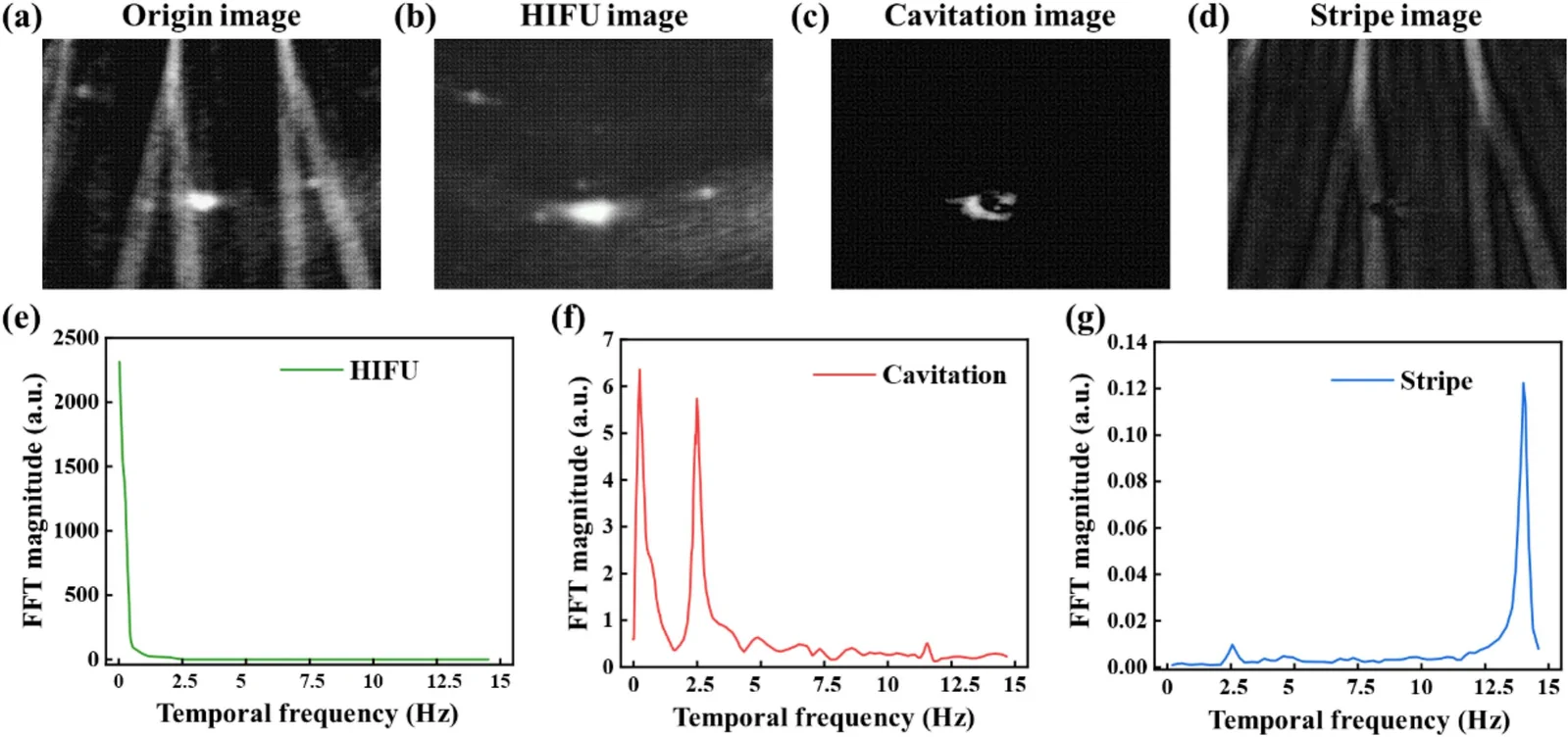
Representative separation results of the SVD-based filtering method for a single frame. (a) Original B-mode image. (b) HIFU driving component. (c) Cavitation component. (d) Stripe-interference component. (e-g) Temporal amplitude spectra of the representative image-derived components associated with panels (b-d). The spectra were calculated from 120 consecutive frames acquired at 30 frames/s, with a frequency-bin spacing of 0.25 Hz. The displayed FFT bins span 0–14.75 Hz, and the horizontal axes extend to the Nyquist limit of 15 Hz. The vertical axes show FFT magnitude in arbitrary units.

**Fig. 4:**
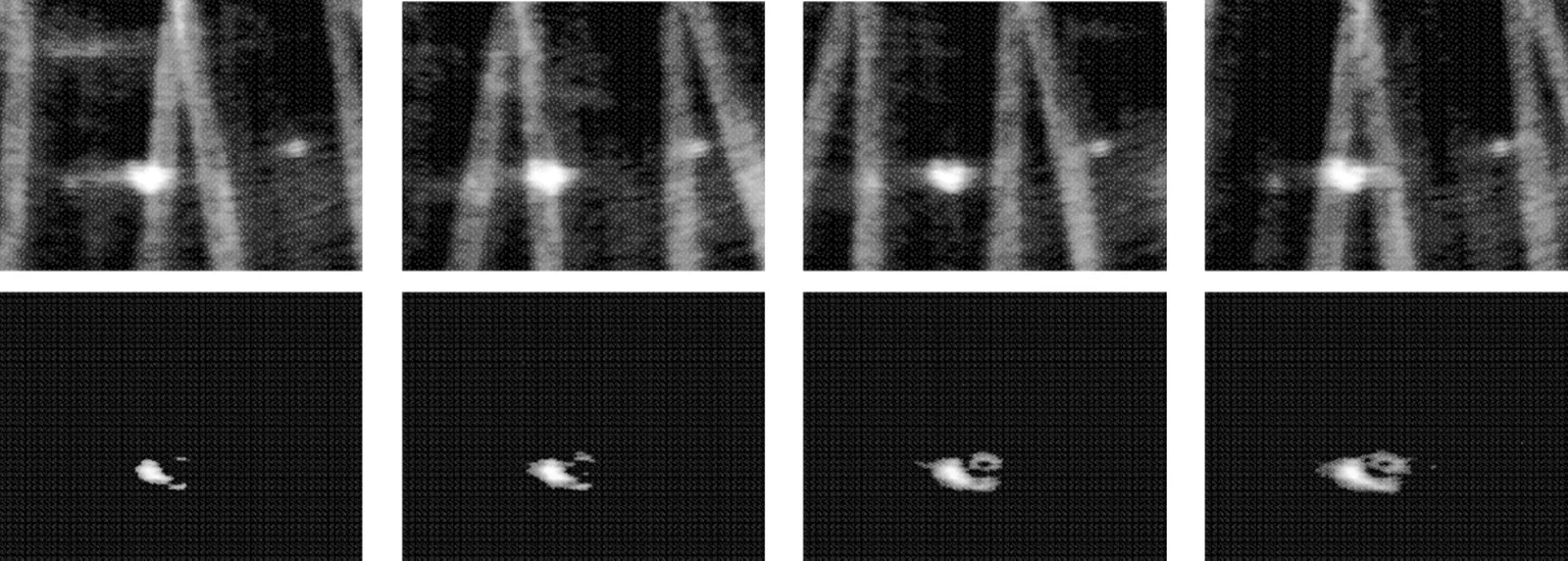
Temporal examples at four consecutive time points. The top row shows the original B-mode images, and the bottom row shows the SVD-processed cavitation-only images. The cavitation region progressively expands over time and extends along the beam direction, exhibiting an accumulation-coalescence pattern, while stripe artifacts remain suppressed in the separated results.

These observations suggest that, within the investigated exposure range, cavitation activity evolves with cumulative exposure and progressive bubble-cloud coalescence as insonation continues. They also indicate that the SVD-based spatiotemporal unmixing remains stable on a frame-by-frame basis. No ROI selection or manual parameter tuning is required. Stripe leakage is suppressed throughout the sequence while the cavitation-related component is preserved, providing consistent frame-wise inputs for retrospective area-time analysis.

Previous studies have reported that increased echogenicity in B-mode images can accompany HIFU-induced cavitation [41], [42], [43]. Accordingly, the hyperechoic features retained after SVD-based separation were quantified in pixel units and are referred to here as the cavitation area. Fig. 5 presents the temporal evolution of this metric in gel phantoms and *ex vivo* liver tissue under different acoustic conditions. In this analysis, the onset of a cavitation-area curve denotes the first detectable rise of the image-derived metric. Fig. 5 presents the time-resolved evolution of the pixel-based cavitation area in both gel phantoms and *ex vivo* liver tissue under different acoustic conditions. Two parameter pathways were investigated: variation of pulse length at fixed acoustic pressure (Figs. 5a and 5c) and variation of acoustic pressure at fixed pulse length (Figs. 5b and 5d). These curves were used to compare cavitation onset, early growth, and late-stage stabilization across exposure settings.

**Fig. 5:**
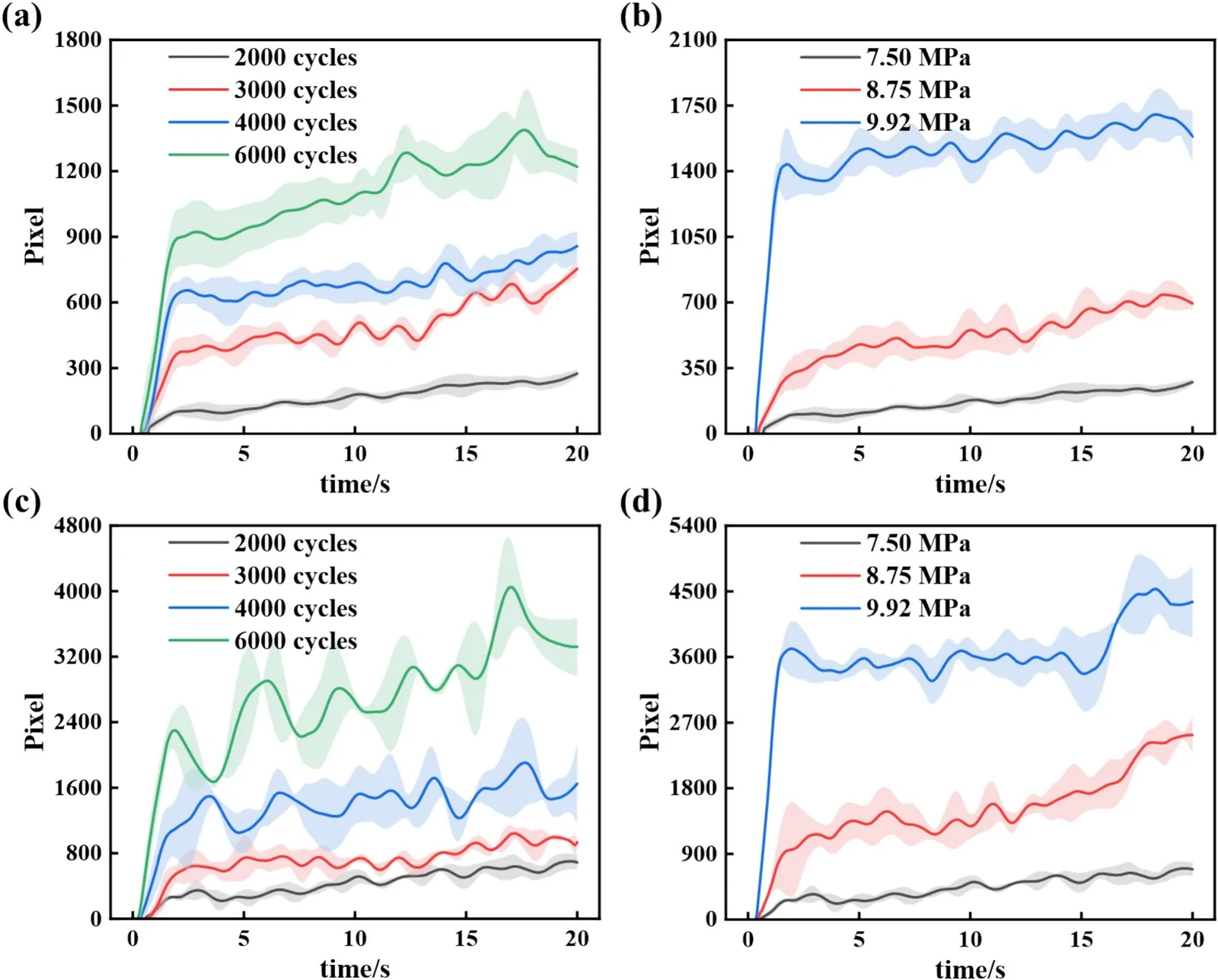
Time courses of the cavitation area under different acoustic conditions. (a) Gel phantom: cavitation evolution at fixed acoustic pressure (7.5 MPa) with varying pulse lengths. (b) Gel phantom: cavitation evolution at fixed pulse length (2000 cycles) with varying acoustic pressures. (c) *Ex vivo* liver tissue: cavitation evolution at fixed acoustic pressure (7.5 MPa) with varying pulse lengths. (d) *Ex vivo* liver tissue: cavitation evolution at fixed pulse length (2000 cycles) with varying acoustic pressures.

In the gel phantom, when the acoustic pressure was fixed at 7.50 MPa, longer pulse lengths produced earlier and steeper increases in the cavitation-area curves (Fig. 5a). The curves rose rapidly during the initial phase and then gradually approached a more stable level, with longer pulse lengths corresponding to higher late-stage plateau values.

When the pulse length was fixed at 2000 cycles, increasing acoustic pressure shifted the cavitation-area curves toward earlier onset, faster initial growth, and higher late-stage values in the gel phantom (Fig. 5b).

In *ex vivo* liver tissue, increasing pulse length at 7.50 MPa produced similar time-dependent changes, with higher cavitation-area curves and more evident late-stage separation among pulse-length conditions (Fig. 5c). Compared with the gel phantom, the liver tissue showed larger image-derived cavitation areas under the same pressure setting.

Likewise, when the pulse length was fixed at 2000 cycles, increasing acoustic pressure in *ex vivo* liver tissue also advanced the onset of the cavitation-area curves and increased their early growth rate and late-stage level (Fig. 5d). The separation among pressure conditions was more evident in liver tissue than in the gel phantom.

Together, these curves show that acoustic pressure mainly affected cavitation onset and initial area, whereas pulse length mainly affected persistence and final stabilized area. The area-time curves provided the basis for subsequent comparisons of cavitation-area distributions and post-exposure morphology.

## 4 Discussion

The present study introduces an SVD-based cavitation-separation framework for active B-mode ultrasound and evaluates its utility as an image-based cavitation area-time quantification tool. The key contribution is not simply artifact suppression, but the conversion of structurally contaminated B-mode sequences into full-field cavitation maps from which onset, growth, persistence, and exposure-dependent area evolution can be quantified. In this sense, the proposed method provides a practical bridge between qualitative visualization of hyperechoic regions and a more structured description of cavitation dosimetry.

### 4.1 Comparison between the SVD method and the grayscale-threshold method

The SVD method was compared with conventional grayscale thresholding under three representative acoustic conditions (Fig. 6). Temporal performance was evaluated from the feature-area-time curves produced by the two image-processing methods. In all tested cases, the SVD-derived curves began to rise earlier and showed larger early-stage image responses than the grayscale-derived curves. These findings demonstrate greater sensitivity to weak image features after suppression of structured interference.

**Fig. 6:**
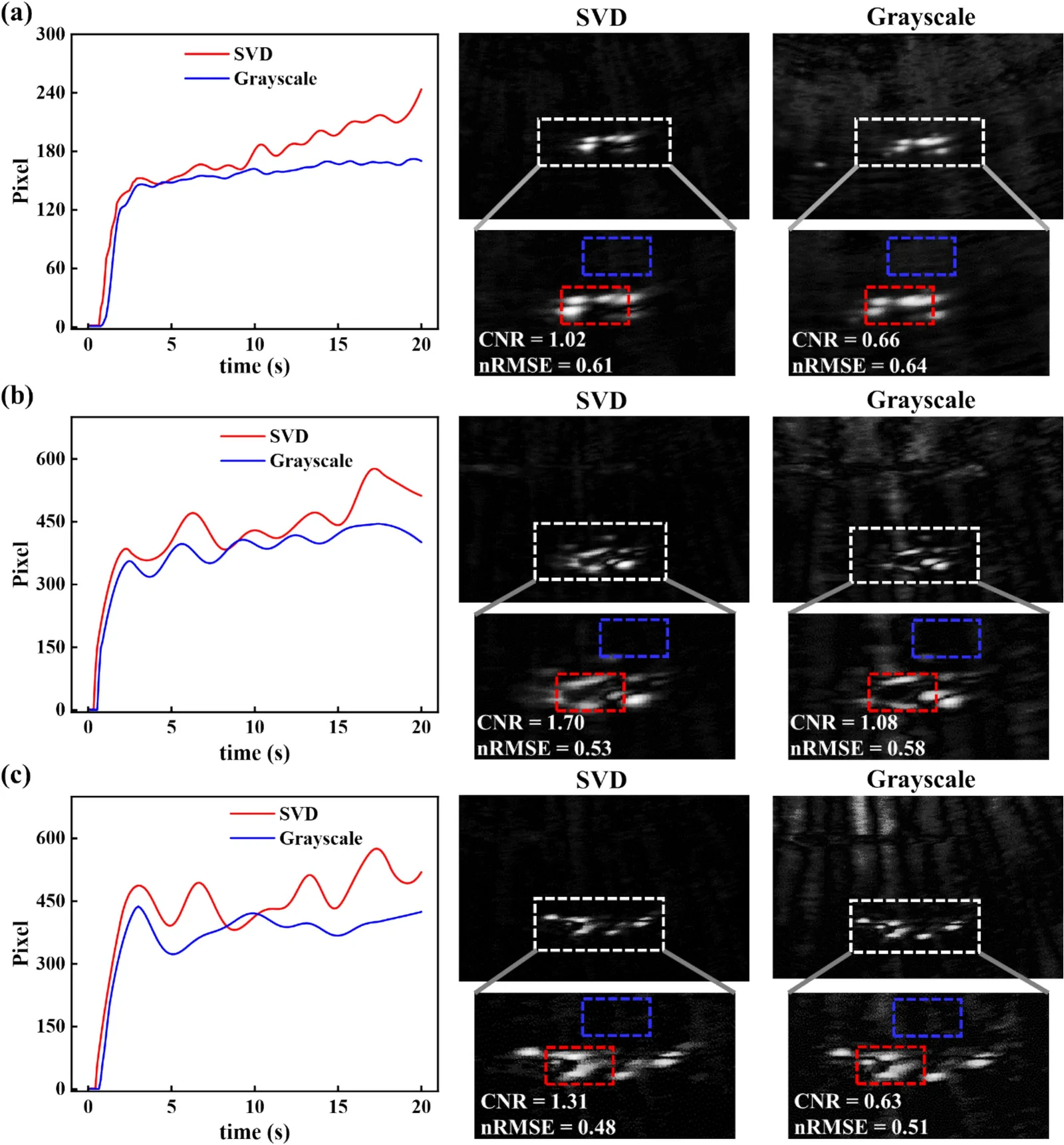
Left: time evolution of cavitation area obtained using the SVD method and the grayscale-threshold method. Right: late-stage cavitation feature images processed by the SVD method and the grayscale-threshold method. The cavitation region is indicated by the white dashed box. In the partial enlarged view, ROIs used for quantitative analysis are marked as cavitation feature regions (red boxes) and fringe-noise regions (blue boxes). CNR was calculated between these two ROIs, and nRMSE was defined as the residual RMS intensity in the fringe-noise ROI normalized by the RMS intensity in the cavitation ROI. (a) 7.50 MPa, 2000 cycles. (b) 8.75 MPa, 2000 cycles. (c) 7.50 MPa, 4000 cycles.

At 7.50 MPa and 2000 cycles, the SVD-based curve increased several frames earlier than the grayscale-based curve and remained at a higher level during the subsequent 20 s observation window (Fig. 6a). The same behavior was preserved when the pressure was increased to 8.75 MPa (Fig. 6b) and when the pulse length was increased to 4000 cycles at fixed pressure (Fig. 6c). This consistent lead in onset detection suggests that the SVD-based framework is more responsive to weak or early-stage cavitation activity and remains sensitive as the acoustic dose increases.

Image quality and extraction performance were further quantified by the contrast-to-noise ratio (CNR) and the normalized root-mean-square error (nRMSE), following Zhu et al. [44]. CNR was used to quantify the contrast between the cavitation region and the fringe-noise region, whereas nRMSE was used as a normalized residual-artifact metric to evaluate the residual signal leakage in the fringe-noise region relative to the cavitation signal. Using the cavitation region, background tissue, and fringe-noise region as reference ROIs, the SVD method consistently produced higher CNR and lower nRMSE than grayscale thresholding in all tested conditions (Table 1; Fig. 6), confirming that the improvement was not limited to qualitative visualization but also reflected enhanced quantitative separability.

**Table 1:** Comparison of cavitation-fringe contrast and normalized residual artifact level between the SVD method and grayscale-threshold method under different acoustic conditions.

Acoustic condition	CNR (SVD)	CNR (Grayscale)	nRMSE (SVD)	nRMSE (Grayscale)
7.50 MPa/2000 cycles	1.02	0.66	0.61	0.64
8.75 MPa/2000 cycles	1.70	1.08	0.53	0.58
7.50 MPa/4000 cycles	1.31	0.63	0.48	0.51

The visual results agreed with these metrics. SVD reduced residual energy in fringe-artifact regions, sharpened cavitation boundaries and yielded a more homogeneous background. Grayscale thresholding was more vulnerable to underestimation and false detection when signal-to-noise ratio was low or stripe artifacts dominated the image.

Considering both temporal response and image-quality performance, the SVD method provided more consistent extraction of candidate cavitation-associated image features than grayscale thresholding under the tested conditions. The improvement is consistent with the ability of spatiotemporal rank decomposition to suppress coherent interference while preserving short-lived, nonstationary image fluctuations. The higher CNR and lower nRMSE demonstrate improved image-domain separability; they do not by themselves verify the physical origin of the reconstructed signals. The SVD-derived area can therefore be used as a relative image-based variable for comparison among exposure settings, while synchronized acoustic measurements would provide additional direct calibration of cavitation emissions.

For low-latency and robust online monitoring, the SVD-based method was therefore more reliable than conventional grayscale discrimination. The resulting area-time curves can be compared with post-exposure axial damage observations to interpret exposure-dependent cavitation evolution. Within the tested frame rate and sample conditions, the method was better suited than grayscale discrimination for low-latency cavitation quantification in contaminated B-mode sequences.

### 4.2 Effect of pulse length on cavitation area in B-mode images

At fixed acoustic pressure, pulse length produced a consistent and reproducible modulation of cavitation extent. As summarized in Fig. 7, the cavitation area increased monotonically with pulse length in both the gel phantom and *ex vivo* liver tissue, indicating a clear dose dependence of the SVD-derived metric with respect to exposure duration.

**Fig. 7:**
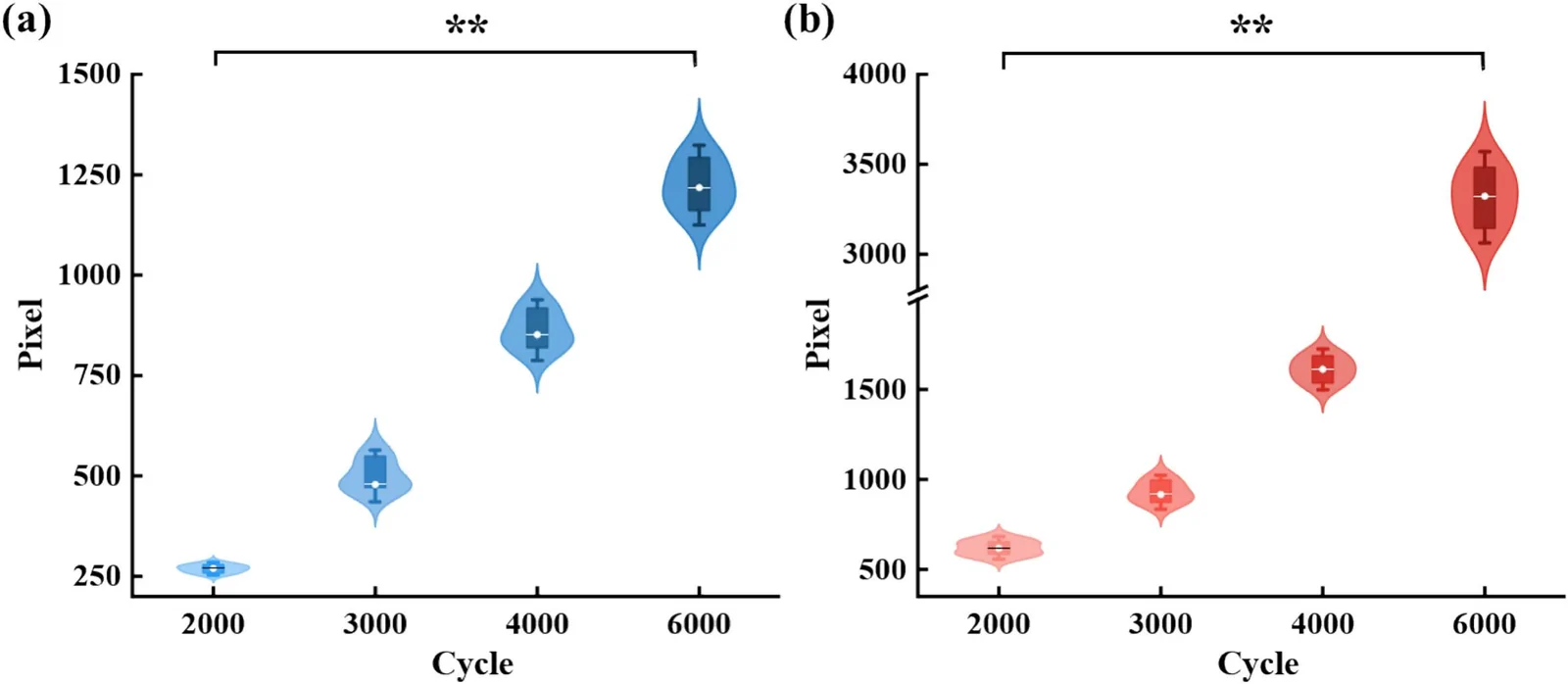
Statistical distributions of cavitation area under four pulse-length conditions (2000–6000 cycles). (a) Gel phantom. (b) *Ex vivo* liver tissue. ** indicates statistically significant differences between groups (p < 0.01).

In the gel phantom at 7.5 MPa, both the median cavitation area and the distribution width increased as the pulse length was extended from 2000 to 6000 cycles (Fig. 7a). Over this range, the cavitation area expanded from approximately 250 pixels to more than 1500 pixels. This trend suggests that, at constant pressure, longer pulse trains sustain bubble activity for a longer period and thereby promote progressive spatial growth of the cavitation region. The effect became more evident at higher cycle numbers, consistent with a cumulative-exposure mechanism.

In *ex vivo* liver tissue at 7.5 MPa, cavitation area also increased with pulse length and remained larger than that in the gel phantom (Fig. 7b). The median area increased from approximately 1200 pixels to nearly 4000 pixels, with steeper growth at higher cycle numbers. The marked rise between 4000 and 6000 cycles may indicate a pulse-length-dependent activation threshold or nonlinear amplification. In tissue, longer exposure may also promote more continuous axial disruption [45], [46], [47].

Overall, these results indicate that pulse length primarily governs the persistence and cumulative enlargement of the cavitation region. Within the tested range, the SVD-derived cavitation area captured this exposure dependence in a stable manner, supporting its suitability for dose-response analysis based on treatment duration.

### 4.3 Effect of acoustic pressure on cavitation area in B-mode images

At fixed pulse length, acoustic pressure exerted a strong and systematic effect on cavitation extent. Fig. 8 shows that cavitation area increased monotonically with pressure in both the gel phantom and *ex vivo* liver tissue, demonstrating that the proposed metric is also sensitive to variations in instantaneous acoustic driving strength.

**Fig. 8:**
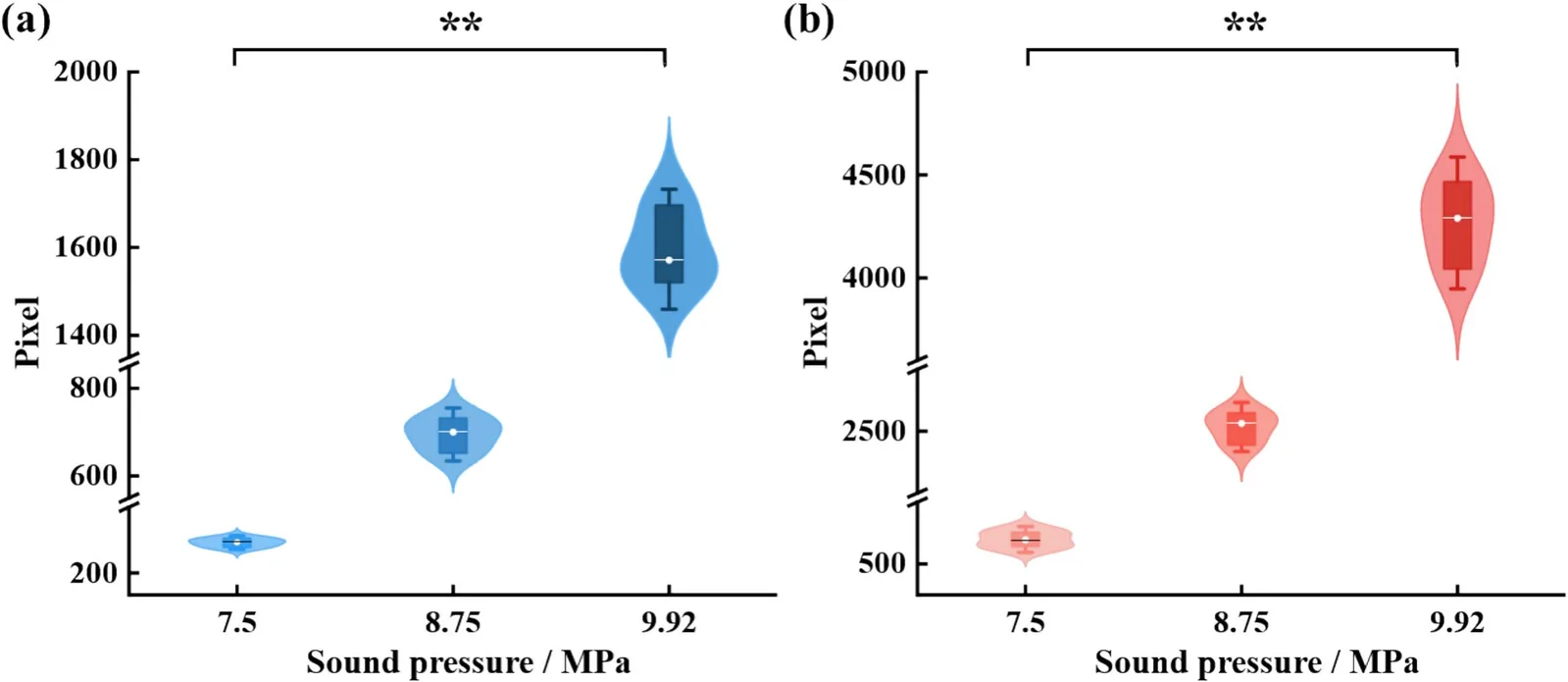
Statistical distributions of cavitation area under three acoustic-pressure conditions (7.5, 8.75, and 9.92 MPa). (a) Gel phantom. (b) *Ex vivo* liver tissue. ** indicates statistically significant differences between groups (p < 0.01).

In the gel phantom at 2000 cycles, increasing pressure from 7.5 to 9.92 MPa increased the median cavitation area from approximately 270 pixels to more than 900 pixels, while also broadening the distribution (Fig. 8a). This behavior is consistent with pressure-dependent recruitment of cavitation activity, in which higher peak negative pressure facilitates bubble nucleation, accelerates cavitation onset, and supports a larger spatial extent of the cavitating region [48], [49], [50].

In *ex vivo* liver tissue, the pressure effect was even more pronounced (Fig. 8b). Higher pressure led to larger detected areas, and the distribution became increasingly right-skewed at high pressure. Relative to the gel phantom, the steeper growth in tissue suggests that acoustic nonlinearity, absorption, and microstructural heterogeneity amplify the pressure dependence of cavitation-induced damage. This is consistent with the stronger cavitation response observed in tissue throughout the study.

Fig. 7, Fig. 8 together show that both pulse length and acoustic pressure modulate cavitation area. Higher pressure mainly advanced cavitation onset and increased the initial extent, whereas longer pulses further enlarged the region through cumulative exposure. The fact that these two effects were captured consistently by the SVD-based metric supports its utility for parameter-sensitive cavitation dosimetry under active B-mode monitoring conditions.

### 4.4 Effect of pulse length on cavitation-induced damage

To further examine the influence of pulse length on cavitation-induced damage, experiments were performed at a fixed acoustic pressure of 7.5 MPa using four pulse lengths (2000, 3000, 4000, and 6000 cycles). Morphological changes are presented in Fig. 9 as paired views of two-dimensional axial cross-sections and corresponding three-dimensional reconstructions for the gel phantom (Fig. 9a) and *ex vivo* liver tissue (Fig. 9b). As stated in the Methods, the three-dimensional volume readout was used only to reflect relative changes in damage extent across conditions and to support interpretation of the two-dimensional cavitation-area evolution, rather than to provide absolute geometric or histological ground truth.

**Fig. 9:**
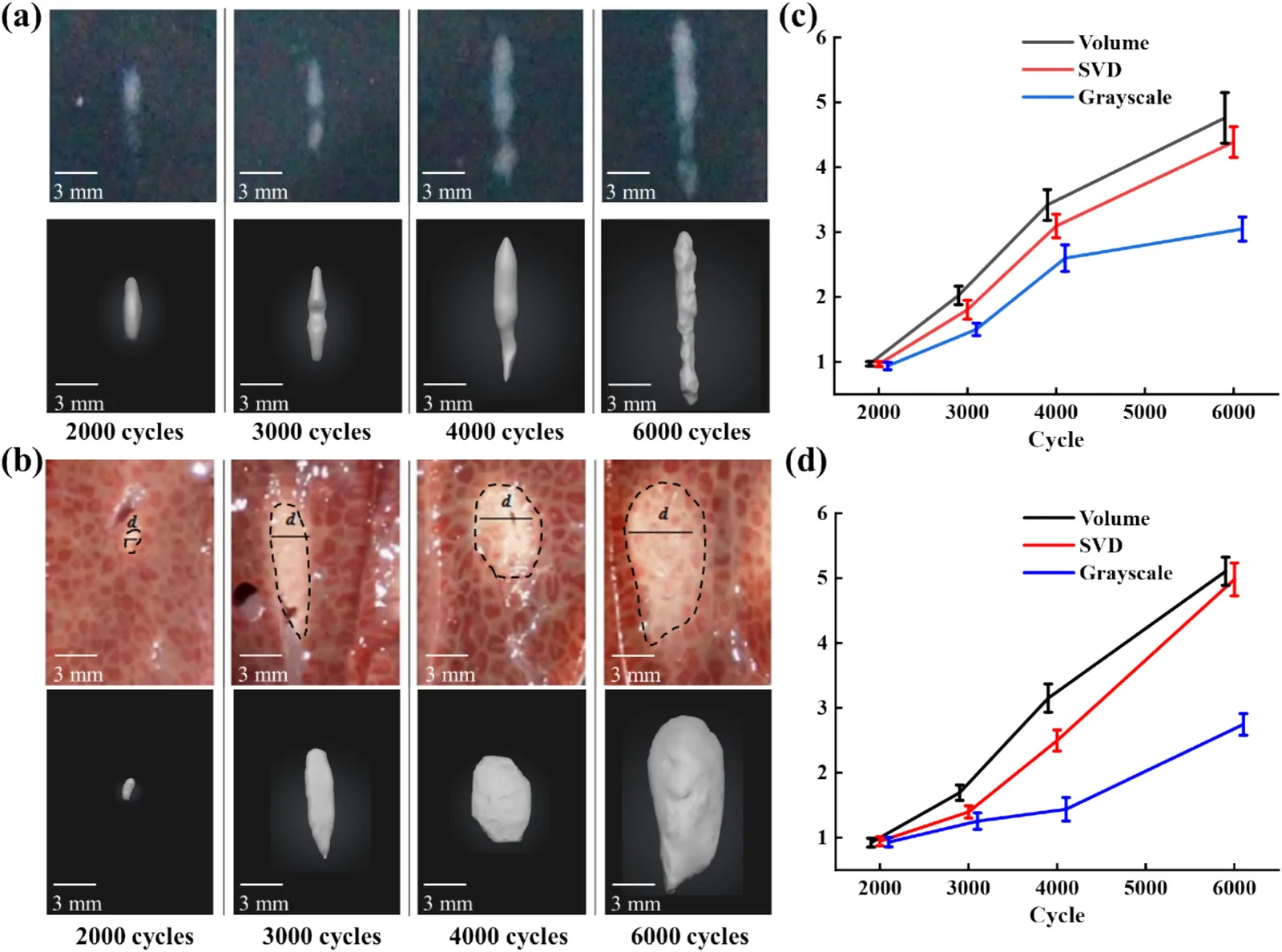
Cavitation-induced damage at a fixed acoustic pressure of 7.5 MPa with pulse lengths of 2000, 3000, 4000, and 6000 cycles. Paired views are shown as two-dimensional axial cross-sections and the corresponding three-dimensional visual renderings of damage morphology. (a) Gel phantom. (b) *Ex vivo* liver tissue. (c) Gel phantom: damage observations with cavitation area estimated by the SVD method and the grayscale-threshold method. (d) *Ex vivo* liver tissue: damage observations with cavitation area estimated by the SVD method and the grayscale-threshold method.

In the gel phantom, increasing the pulse length from 2000 to 6000 cycles changed the damage pattern from short and localized hyperechoic spots to more continuous and axially elongated streak-like structures (Fig. 9a). The reconstructed volume increased accordingly, and axial elongation became progressively more apparent. These findings suggest that prolonged exposure not only enlarges the cavitation region but also promotes morphological consolidation of the affected zone.

In *ex vivo* liver tissue, the damage pattern at 2000 cycles was limited and morphologically irregular (Fig. 9b). With increasing cycle number, both damage depth and cross-sectional area increased, and the cavitation region expanded progressively. At 4000-6000 cycles, the lesion boundary became clearer and the overall morphology more complete. This progression was in good agreement with the corresponding increase in the SVD-derived cavitation area, supporting internal consistency between the dynamic metric and the morphological endpoint.

Figs. 9c and 9d compare cavitation-area estimates obtained by SVD and grayscale thresholding across pulse-length conditions. SVD captured the growth trend in both media and yielded larger area estimates. The difference became more evident at 4000 and 6000 cycles, suggesting improved tracking of prolonged cavitation activity.

These observations support the area-time analysis. Longer pulse trains increased both the spatial extent and morphological continuity of cavitation-induced damage, while the SVD method provided stable separation and quantification across the tested pulse lengths.

### 4.5 Effect of acoustic pressure on cavitation-induced damage

To evaluate the pressure dependence of cavitation-induced damage, the pulse length was fixed at 2000 cycles. Experiments were conducted in a gel phantom and *ex vivo* liver tissue at three acoustic pressures (7.5, 8.75, and 9.92 MPa). The extent of the cavitation region showed a strong pressure dependence (Fig. 10). Figs. 10a and 10b present two-dimensional axial cross-sections together with the corresponding three-dimensional reconstructions for the gel phantom and *ex vivo* liver tissue, respectively.

**Fig. 10:**
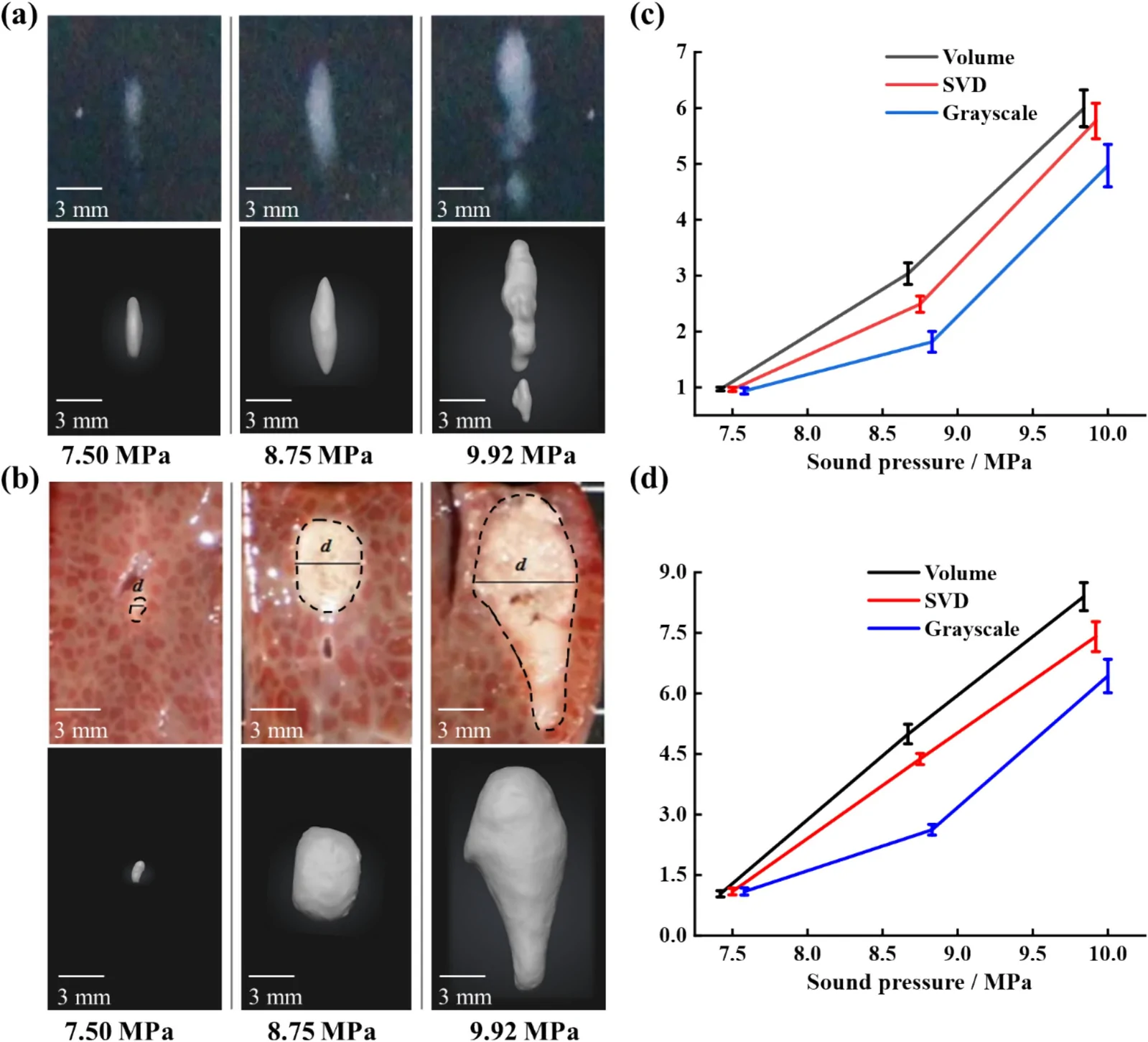
Cavitation-induced damage at a fixed pulse length of 2000 cycles and acoustic pressures of 7.5, 8.75, and 9.92 MPa. Paired views are shown as two-dimensional axial cross-sections and the corresponding three-dimensional visual renderings of damage morphology. (a) Gel phantom. (b) *Ex vivo* liver tissue. (c) Gel phantom: damage observations with cavitation area estimated by the SVD method and the grayscale-threshold method. (d) *Ex vivo* liver tissue: damage observations with cavitation area estimated by the SVD method and the grayscale-threshold method.

In the gel phantom, damage at 7.5 MPa was limited and morphologically irregular (Fig. 10a). As pressure increased to 8.75 and 9.92 MPa, the reconstructed damage volume increased rapidly. At 9.92 MPa, both compact clusters and slender column-like structures were observed, suggesting coexistence of intense localized cavitation near the focus and axial channel-like extension. Compared with the changes induced by pulse-length variation, pressure produced a larger incremental increase in damage extent and also introduced greater morphological variability. This indicates that pressure is a dominant determinant of cavitation initiation intensity and of the resulting damage severity, and therefore must be selected with particular attention to the balance between treatment efficiency and safety.

A similar but stronger trend was observed in *ex vivo* liver tissue (Fig. 10b). With increasing pressure, the cavitation region expanded radially, and both damage depth and cross-sectional area increased. At the highest pressure, the damage became more severe and channel-like disruption was more evident. This behavior suggests that higher pressure not only promotes cavitation-bubble formation but also aggravates tissue disruption, likely through repeated bubble collapse, cloud clustering, and enhanced local mechanical interaction.

Comparison between the SVD method and grayscale thresholding under different pressure conditions (Figs. 10c and 10d) again showed that the SVD method yielded larger cavitation-associated area estimates and a more coherent pressure-dependent trend in both media. Importantly, the pressure-dependent enlargement of the SVD-derived regions was consistent with the increasing extent and severity of the directly observed post-exposure damage, providing independent morphological corroboration of the exposure-dependent trend captured by the proposed method. These findings support the view that improved cavitation-associated feature quantification is achieved through signal-component separation rather than through adjustment of grayscale thresholds alone. In addition, the stable performance at higher pressures suggests that the method remains robust under conditions with stronger cavitation-associated image changes and more severe image contamination.

Acoustic pressure affected cavitation-induced damage through both expansion of the affected region and morphology changes. With increasing pressure, cavitation area increased, and damage became deeper and more complex. Across all pressure conditions, the SVD-based method provided stable separation and quantification of cavitation regions under the tested settings.

### 4.6 Limitations

In the proposed framework, singular components were selected using both singular-value magnitude and temporal spectral distribution. This approach suppressed the stripe patterns observed in the present recordings. However, interference components may also satisfy the selection criterion when their sampled temporal spectra overlap the accepted band. This includes stripe periodicity, mains interference, and PRF-related fluctuations, whose observed spectral content may be affected by sampling, aliasing, and modulation. The observed suppression therefore applies to the conditions examined here; performance under controlled spectral-overlap conditions and varied PRF remains to be established.

The current batch-processing implementation reduces the need for manual cavitation-region outlining, but it does not demonstrate real-time causal processing. Because each SVD reconstruction was obtained from a finite frame stack, the reported area-time curves should be interpreted as retrospective image-domain measurements rather than online detection outputs.

Several limitations remain. First, because the analysis was performed on log-compressed B-mode images, the reconstructed components and segmented areas should be interpreted as image-derived metrics. They should not be interpreted as calibrated acoustic pressure, acoustic energy, or absolute cavitation dose. Second, computational burden increases with the number of frames, and GPU parallelization or multiscale SVD may further reduce latency. A real-time implementation will require a defined sliding-window strategy, causal component selection, and direct latency testing on the target hardware. Third, boundary ambiguity persists in some cavitation regions and may be improved by refined post-processing or learning-based segmentation. Fourth, synchronized PCD or hydrophone measurements were not acquired for the present B-mode dataset. Nevertheless, previous studies have reported a close correspondence between HIFU-induced B-mode hyperechogenicity and cavitation activity using acoustic cavitation detection [23], [41]. In the present experiments, the samples were additionally sectioned after HIFU exposure and the macroscopic damage morphology was photographically documented, providing an independent morphological endpoint for comparison with the B-mode-derived measurements. Future studies incorporating synchronized PCD or passive acoustic mapping may further establish the temporal correspondence between acoustic emissions and the proposed image-domain metric. Robustness should be evaluated in vivo under physiological motion and perfusion. Multimodal fusion with temperature readout or shear-wave elastography may also improve interpretation [46], [51], [52]. Finally, a dose model combining cavitation time, area and acoustic pressure may further support quantitative exposure planning.

## 5 Conclusion

In this study, We developed an SVD-based framework for separating cavitation-related signals from structured artifacts in active B-mode ultrasound sequences. By exploiting differences in spatiotemporal coherence and temporal spectral-energy distribution, the method reconstructed full-field cavitation maps and enabled continuous area-time tracking without a predefined ROI.

Experiments in gel phantoms and *ex vivo* liver tissue showed that the extracted area increased with both pulse length and acoustic pressure, and these exposure-dependent trends were consistent with directly observed post-exposure damage morphology. The proposed framework therefore provides an interpretable image-domain approach for spatiotemporal assessment and relative comparison of HIFU-induced cavitation-associated changes. Future synchronized PCD or passive acoustic mapping may further provide direct acoustic calibration of the image-domain metric.

The proposed readout provides an interpretable image-based metric for retrospective spatiotemporal assessment and relative comparison of cavitation-associated features derived from active B-mode sequences. The observed feature-area-time trends may support parameter comparison and motivate future online monitoring studies, whereas real-time feedback use will require a separately implemented and timed causal pipeline.

Further work should evaluate robustness using no-HIFU controls, systematic variation of PRF and imaging frame rate, and controlled periodic-interference conditions, particularly when the sampled interference spectrum overlaps the accepted band. Prospective studies incorporating synchronized passive cavitation detection, hydrophone measurements, or passive acoustic mapping would also help establish the physical specificity of the reconstructed image-domain feature.

## CRediT authorship contribution statement

**Xinyue Gong:** Writing – original draft, Investigation, Data curation. **Zijun Liu:** Investigation, Data curation. **Guofeng Zhang:** Validation, Software, Data curation. **Shuyuan Gu:** Data curation. **Yifei Zhu:** Software. **Honghui Xue:** Visualization. **Qinsong Xu:** Writing – review & editing. **Jun Wu:** Writing – review & editing, Project administration. **Juan Tu:** Writing – review & editing, Writing – original draft, Resources.

## Declaration of Generative AI and AI-assisted technologies in the writing process

During the preparation of this work, the authors used ChatGPT to assist with language editing and to improve manuscript readability. After using this tool, the authors carefully reviewed and edited the content as needed and take full responsibility for the final published version.

## Declaration of competing interest

The authors declare that they have no known competing financial interests or personal relationships that could have appeared to influence the work reported in this paper.

## Appendix A Supplementary data

Supplementary Data 1 Supplementary material: Sensitivity analysis of spectral-gating parameters and an algorithmic stress test of spectral overlap using deterministic sampled sinusoids (Figs. S1 and S2).
