# Supplementary material for: Image-domain assessment and quantitative evaluation of acoustic cavitation using singular-value decomposition and magnitude-based spectral gating of B-mode imaging
Source: Ultrason Sonochem. 2026 Oct 13;134:108114. doi: 10.1016/j.ultsonch.2026.108114 (PMC13695313; doi:10.1016/j.ultsonch.2026.108114)
Supplement: Supplementary file 1 [file mmc1.docx]

Supplementary

Parameter sensitivity analysis

The direct gating response and the downstream reconstructed image response were evaluated separately. With the accepted band fixed at *k* = 1, 2 and 3, increasing the magnitude-fraction threshold from 0.056 to 0.084 progressively reduced or preserved the number of retained singular components in all three archived sequences (Fig. S1a), consistent with the selection rule $A_{i}>\tau$. Conversely, with $\tau=0.07$ fixed, increasing the highest included non-DC bin from $q=2$to $q=5$increased or preserved the number of retained components (Fig. S1b).

The corresponding reconstructed foreground-pixel measurements showed additional sequence-dependent changes (Figs. S1c and d). Because this quantity was calculated after component reconstruction and image-domain segmentation, it represents a downstream image readout rather than the gating decision itself and therefore does not necessarily vary monotonically with the retained-component count. These results indicate that the reference setting, $\tau=0.07$ and $q=3$, provides an operational parameter set for the present sequences, while further calibration against an independent cavitation reference would be required before treating these parameters as universally optimized.

**Fig. S1.** Retrospective sensitivity analysis of the magnitude-based spectral gate. (a) Retained singular-component count as a function of the magnitude-fraction threshold *τ*, with the accepted non-DC bins fixed at *k* = 1, 2 and 3. (b) Retained singular-component count as a function of the highest included non-DC bin q, with *τ*=0.07 fixed. (c) Corresponding reference-normalized mean foreground pixel count for the threshold-sensitivity analysis. (d) Corresponding reference-normalized mean foreground pixel count for the included-bin analysis. The reference setting was *τ*=0.07 and *q*=3 (*k* = 1, 2 and 3, 0.25-0.75 Hz). Foreground-pixel measurements were normalized to the corresponding value obtained with this reference setting for each archived sequence. The y-axis in panel (d) is shown on a base-2 logarithmic scale. Panels (a) and (b) characterize the direct response of the spectral gate, whereas panels (c) and (d) show the downstream image-domain response after component reconstruction and foreground segmentation.

Algorithmic stress test for spectral overlap

To examine the limiting case of spectral overlap, the same gating rule was applied to deterministic sampled sinusoids. Using $N=120$, $f_{s}=30$ frames/s, accepted non-DC bins *k* = 1, 2 and 3, and a threshold of 0.07, sinusoids whose frequencies coincided with the accepted bins readily satisfied the gating criterion. Frequencies adjacent to the accepted band could also exceed the threshold because of finite-window spectral leakage. This test demonstrates that periodic interference may be retained when its sampled temporal spectrum overlaps the accepted band.

**Fig. S2.** Response of the spectral gate to deterministic sampled sinusoids over the file Nyquist interval. The dashed line indicates the gating threshold of 0.07. Sinusoids within or near the accepted bins can pass the gate because of direct spectral overlap or finite-window leakage. This calculation illustrates an algorithmic overlap condition and is not an ultrasound interference experiment.
